# A comprehensive scheme for the objective upper body assessments of subjects with cerebellar ataxia

**DOI:** 10.1186/s12984-020-00790-3

**Published:** 2020-12-04

**Authors:** Ha Tran, Khoa D. Nguyen, Pubudu N. Pathirana, Malcolm K. Horne, Laura Power, David J. Szmulewicz

**Affiliations:** 1 School of Engineering, Deakin University, Pigdons Road, Waurn Ponds, VIC 3220 Australia; 2 Florey Institute of Neuroscience and Mental Health, Royal Parade, Parkville, VIC 3052 Australia; 3 Balance Disorders & Ataxia Service, Royal Victorian Eye and Ear Hospital (RVEEH), Gisborne St, East Melbourne, VIC 3002 Australia; 4 Cerebellar Ataxia Clinic, Alfred Hospital, Commercial Road, Prahran, VIC 3004 Australia

**Keywords:** Cerebellar ataxia, Finger chase, Finger tapping, Finger to nose, Dysdiadochokinesia, Objective assessment, Feed backward feature elimination

## Abstract

**Background:**

Cerebellar ataxia refers to the disturbance in movement resulting from cerebellar dysfunction. It manifests as inaccurate movements with delayed onset and overshoot, especially when movements are repetitive or rhythmic. Identification of ataxia is integral to the diagnosis and assessment of severity, and is important in monitoring progression and improvement. Ataxia is identified and assessed by clinicians observing subjects perform standardised movement tasks that emphasise ataxic movements. Our aim in this paper was to use data recorded from motion sensors worn while subjects performed these tasks, in order to make an objective assessment of ataxia that accurately modelled the clinical assessment.

**Methods:**

Inertial measurement units and a Kinect© system were used to record motion data while control and ataxic subjects performed four instrumented version of upper extremities tests, i.e. finger chase test (FCT), finger tapping test (FTT), finger to nose test (FNT) and dysdiadochokinesia test (DDKT). Kinematic features were extracted from this data and correlated with clinical ratings of severity of ataxia using the Scale for the Assessment and Rating of Ataxia (SARA). These features were refined using Feed Backward feature Elimination (the best performing method of four). Using several different learning models, including Linear Discrimination, Quadratic Discrimination Analysis, Support Vector Machine and K-Nearest Neighbour these extracted features were used to accurately discriminate between ataxics and control subjects. Leave-One-Out cross validation estimated the generalised performance of the diagnostic model as well as the severity predicting regression model.

**Results:**

The selected model accurately ($96.4\%$) predicted the clinical scores for ataxia and correlated well with clinical scores of the severity of ataxia ($rho = 0.8$, $p < 0.001$). The severity estimation was also considered in a 4-level scale to provide a rating that is familiar to the current clinically-used rating of upper limb impairments. The combination of FCT and FTT performed as well as all four test combined in predicting the presence and severity of ataxia.

**Conclusion:**

Individual bedside tests can be emulated using features derived from sensors worn while bedside tests of cerebellar ataxia were being performed. Each test emphasises different aspects of stability, timing, accuracy and rhythmicity of movements. Using the current models it is possible to model the clinician in identifying ataxia and assessing severity but also to identify those test which provide the optimum set of data.

*Trial registration* Human Research and Ethics Committee, Royal Victorian Eye and Ear Hospital, East Melbourne, Australia (HREC Reference Number: 11/994H/16).

## Background

Cerebellar ataxia (CA) describes the dysfunctional balance, gait [1, 2] and limb function [3] that results from cerebellar dysfunction. Ataxia is assessed by observing the performance of standard motor tasks described by Holmes [4, 5] and others almost a century ago. These pioneering clinicians recognised that ataxic movements could not easily be reduced to Newtonian terms but fundamentally manifest as disturbances in accuracy, timing, rhythmicity and stability of the proximal motor platform which they described using terms such as *dysmetria*, *dyssynergia* and *dysrhythmia*.

The standard motor tasks used to assess upper limb ataxia, referred to here as “tests” include the finger chasing test (FCT), finger tapping test (FTT), finger to nose test (FNT) and alternating hand movements looking for dysdiadochokinesia (DDKT) [6, 7]. Scales such as the Scale for the Assessment and Rating of Ataxia (SARA) [6] have been developed to codify the assessment of these tests and require specific aspects of motor dysfunction to be considered when scoring ataxia. For example, the SARA stipulates the overshoot/undershoot distance between subject’s finger and clinician’s finger in the finger chase test. However, there will inevitably be subjectivity and variation in the severity that human observers rate deficits in performance of these tests. The SARA and conventional teaching also recommend administering several tests to characterise upper limb ataxia. However, it is unclear whether this is because each test carries unique information necessary for establishing the presence and severity of ataxia or whether it is because the performance of several different tests provides clinical security despite the redundant information.

Several sensing and information extracting systems have been proposed for quantifying the assessment of upper limb ataxia and thus overcoming subjectivity. For example, a push-button system to evaluate the variation in timing of ataxic movements was considered for the FTT [8–10]. Inertial measurement units (IMUs) have been used to capture movement kinematics in multiple signal domains [11] to objectively assess the FNT [12, 13] and DDKT [12]. The movement of the finger performing the FCT has been tracked using optoelectronic devices ranging from video cameras [14] to VICON [15] and recently Kinect© in our previous study [3] to assess delay in initiating movement and accuracy in reaching the target. While this test identified deficits in accuracy and timing, neither the maintenance of rhythm nor the stability of the execution platform of the moving distal limb were assessed [4]. Thus, it has been possible to emulate individual bed side tests through objective assessments but none of these tests appear to fully assess all aspects of upper limb ataxia (timing, accuracy, rhythmicity and proximal stability). Even in those tests that addressed similar aspects of ataxia, the extent to which they measure the same aspect similarly (i.e. are redundant) is unclear.

In this study, our primary aim is to:

- Develop an Instrumented System for the objective assessment of Upper Limb Ataxia (ISULA). The system includes an IMU sensor module(BioKin™) and Kinect camera to capture movement information from subjects while performing the four conventional tests; namely FCT, FTT, FNT and DDK.
- Identify the minimum combination of tests that provide sufficient information to assess the disability.
- Quantify the heterogeneous aspects intrinsic to ataxia by grouping the extracted features from the system according to clinical domains described by Holmes and others: stability, timing, accuracy, and rhythmicity (referred to here as STAR dimensions).

## Methods

### Participants

Fourteen control subjects (“controls”: mean age, 55; range, 25–68 years) and 41 subjects with cerebellar ataxia (CA or “ataxics”: mean age, 64; range, 28–78 years) participated in this study (Table 1). All ataxics were previously diagnosed with a progressive neurodegenerative ataxia (with genotyping or other confirmatory investigations when relevant, see Table 1—Diagnosis). This study was approved by the Human Research and Ethics Committee, Royal Victorian Eye and Ear Hospital, East Melbourne, Australia (HREC Reference Number: 11/994H/16). Written consent was obtained from all participants.

**Table 1:** Participant demographics

	Controls	Subj. with CA
Total subjects (M/F)	14(5/9)	41(21/20)
Dominant hand, (L/R)	2/12	2/39
Age, mean ± SD (years)	$55 \pm 18$	$64 \pm 15$
SARA score, mean± SD		
Total score	–	$14.23 \pm 9.84$
Upper limb score	–	$3.58 \pm 2.62$
Diagnosis		
CABV/CANVAS	–	8/5
FA/SCAs/Others	–	4/10/14

### Clinical assessments

The severity of ataxia was assessed using SARA [6], on the same day that objective measurements were made. The same clinician, experienced in assessing ataxia, provided all SARA scores to avoid the inevitable scoring variation that occurs when subjects are assessed by different clinicians. SARA assessment is comprised of eight sub-scores: three for the lower limbs (No. 1-2-8), three for the upper limbs (No. 5-6-7), one for sitting (No. 3) and one for speech (No. 4). The SARA scores of upper limb function tests used in this study are: FCT (No. 5), FNT (No. 6) and DDKT (No. 7). Each sub-scores can be scored from 0 to 4 points (5 levels) according to the clinicians assessment of the severity of ataxia when performing the specific test. Hence the upper limb SARA score (SARA-UL) is from 0 to 15 points. The SARA total score (SARA-Total) is calculated by the summation of the eight sub-scores resulting in a maximum of 40 points. In this study, the SARA-Total ($14.23 \pm 9.84$) and the SARA-UL ($3.58 \pm 2.62$) were correlated with the objective assessment of severity of ataxia.

### Automated assessment protocols and apparatus

The ISULA requires the performance of four tests measured by instrumented devices: FCT, FTT, FNT and DDKT. The FCT used a depth sensing camera (Kinect©) to capture the movements of the subject’s finger (while reaching a target on the screen) while in the other three tests, kinematic information was acquired from a 3-dimensional (3D) IMU system, BioKin™ [16] system. The test descriptions and protocols are summarised in Table 2. All the tests were performed under the supervision of an expert clinician (LP). During the test, the clinician wirelessly started and stopped recording and applied markers into the data stream, denoting specific points during the performance through a mobile application. At the end of each trial, the sensor data were uploaded to a cloud-based storage and computing platform for further analysis.

**Fig. 1:**
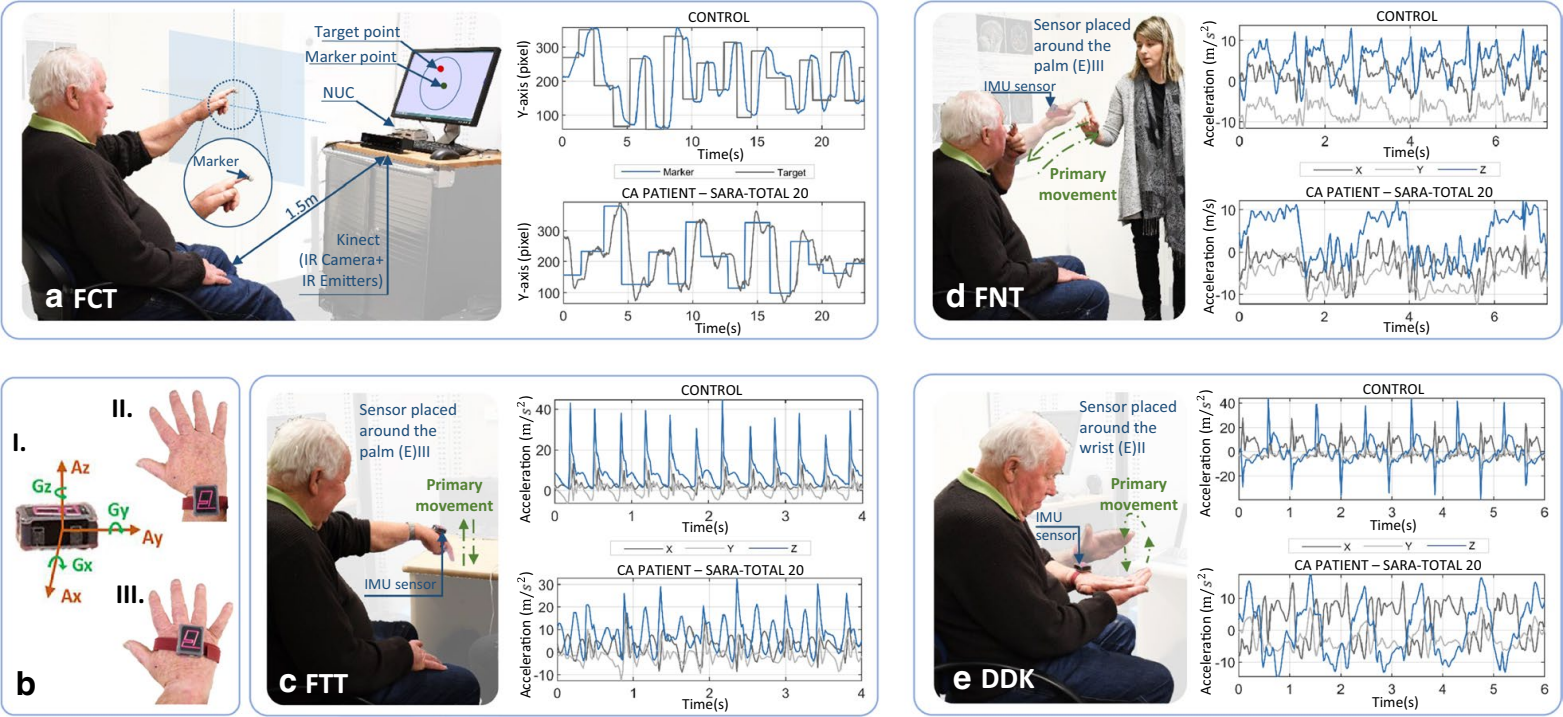
Instrumented version of the upper limb assessments and the movement waveform of a control and a patient diagnosed with CA.** a** Finger Chase (ballistic, FCT) using Kinect© system.** b** I. An IMU sensor with tri-axial accelerometer directions (Ax,Ay,Az) with a gyroscope directions (Gx,Gy,Gz) **b** II. Sensor placement around the wrist **b** III. Sensor placement around the palm. Testing with the IMU system denoting the direction of the primary movement; movement along the direction of effective axis in order to accomplish the task objectives:** c** Finger tapping (FTT), **d** Finger to nose (FNT),** e** dysdiadochokinesia (DDKT)

**Table 2:** Experimental setup and description of tests in ISULA system

Test	Device	Setup	Description
Finger chase test (FCT)	Kinect©	A Microsoft Kinect© V2, a 23 in. screen and a processing computer (Intel core i5) are installed approximately 1.5 m away the subject. The Kinect© captures movements from a 14 mm retro-reflective marker attached on the subject’s index finger. A program randomly generates the target point 20 times on the monitor while projecting the finger movement on the screen	The subject is required to point at and follow a target point on the screen using the index finger. As soon as the projected marker point touches the target point, the target disappears and reappears at a new position. The test is concluded after 20 iterations (Fig. 1a)
Finger tapping test (FTT)	IMU	A sensor was worn on the dorsum of the hand as depicted in Fig. 1(b)III	The subject is required to tap on a tabletop using the index finger at a self-selected and uniform pace. Tapping is performed for approximately 15 s with the elbow and shoulder joints unsupported to assess the stability of the platform (shoulder and elbow) (Fig. 1c)
Finger to nose test (FNT)	IMU	Similar to the setup of finger tapping test	The subject’s index finger moves repeatedly between the the clinician’s finger and the subject’s nose for approximately 15 s. The clinician’s finger is held stationary at a position approximately 50 cm in front of the subject (Fig. 1d)
Dysdiadochokinesia (DDKT)	IMU	A sensor was worn on the wrist as depicted in Fig. 1(b) II	The subject alternates between placing one hand palm-up and palm-down on the other hand as fast and precisely as possible for approximately 10 cycles (Fig. 1e)

### Manifestations of ataxia

Following Holmes [4], we describe four domains of ataxia (using the acronym STAR). The purpose is to develop a system of assessing ataxia that reflect the following generic domains of CA manifestations:

- Stability (S): Lack of stability in the platform during the execution of the task (the oscillations of the movement that is not preferred).
- Timing (T): Error between the goal/time objective against what is achieved in a temporal context. This is likely to be affected by:
  - The time for the subject to initiate a movement.
  - The time to complete a movement/speed.
- Accuracy (A): Error between the goal/space objective against what is achieved in a spatial context.
- Rhythmicity (R): The regularity in repeated movement

### Data preprocessing

Accelerations and angular velocities from the IMU sensor were sampled at 50 Hz in the three orthogonal $X, Y$ and $Z$ axes. These signals were filtered by a 2nd order band-pass Butterworth filter with the cut-off frequency from 0.3 to 20 Hz where the base band frequencies were excluded to minimise drift effects and the high frequencies were restricted to the bandwidth of human movements [17]. In the Kinect© system, the location of each randomly generated instantaneous position change was stored as a pair of position coordinates. The target position remained constant between each change in the target location, while the marker position changed. The Kinect© captured the marker position at a sampling rate of 30 Hz. The maximum frequency of human movement is approximately 20 Hz [17], and for ataxic subjects this can be lower [3]. Especially for peripheral limb motion, the requencies are even less. Therefore, the sampling rate of Kinect is sufficient to capture the motion of subjects in this study.

### Data analysis

Relevant objective measures extracted from each ISULA test were described and associated with the corresponding STAR classification in Table 3. For notational simplicity, the feature names are denoted as: $(FeatureName)^{Axis(L/R)}_{Test}$ with L/R indicating performance by the left or right hand.

#### Finger chase test (FCT)

When assessing the FCT, the clinician subjectively estimates the extent of under/overshooting in the subject’s movements relative to the moving target [6]. The ISULA system automates the assessment of FCT by considering the space–time trajectory of the marker and target. The overshoot/undershoot information of the subject movement was measured by the Dynamic time warping (DTW)-based error. The DTW was used to find the shortest path between the marker $S_m$ and target $S_t$ trajectories via their distance matrix *DS* using dynamic programming

1 $$\begin{aligned} \begin{aligned} DS({i,j}) = dist(S_m(i),S_t(j)) + min\{DS(i-1,j),\\DS(i-1,j-1),DS(i,j-1)\}. \end{aligned} \end{aligned}$$

The error *DTWErr* is calculated by summing the value of the shortest path *P* obtained by going from the last (n; n) to the first (1; 1) element of *DS* via adjacent elements with the smallest values. The time from establishing a new target position to the subject’s initiation of movement was defined as reaction time (*ReTi*). This feature was obtained by cross correlating the two time sequences representing the marker and the target movement.

2 $$\begin{aligned} ReTi = \arg max \left(\sum_{i=-\infty }^{\infty }S_m^*[i]S_t[i+j]\right). \end{aligned}$$

The kinematic delay was obtained from the index of performance measurement described by Fitts’ law [18]. The feature is intended to capture the performance of the subject in reaching a target position outlined by $KiDe = ID/MT$, where $ID=\log _2(di/ra)$ is the index of difficulty of the task while *di* is the distance between the current and previous position of the target, *ra* is the radius of the target circle and *MT* is the execution time of the task by the subject. The acceleration alteration (*AcAlt*) computed the number of times the subjects changed their acceleration while reaching the target. The feature measures the efficiency of force applied to performing the task.

#### Finger tapping test (FTT)

There is greater temporal variability when ataxic subjects tap repetitively than when controls do [19]. This can be observed in the inter-tap interval (ITI) and “movement variability”. The ITI is defined as the duration between successive contacts with the table and its coefficient of variation (*CITI*). This quantifies the variability of the tapping rhythm with respect to the tapping rate [19, 20]. Movement variability is quantified using fuzzy entropy (*FuEn*), obtained for each movement time series (accelerations and angular velocities). Given a N-sample time series $y=\{x_t|^N_{t=1}\}$, *FuEn* defines a states of *m* embedding dimensions such that $X^m_t=\{x_t,x_{t+1},\ldots,x_{t+m-1}\}$ in the phase space and the distance $d_{pq}=d[X^m_p,X^m_q]$ is measured by Chebyshev distance. Instead of using a Heaviside function to count the number of matched pairs of states, the similarity degree $D^m_{pq}$ between any two states ($t=p$ and $t=q$) is quantified using a fuzzy function $D^m_{pq}=exp(-(d_{pq}/r)^2)$ of order 2 and radius *r*. *FuEn* allows variability to be quantified by calculating the reduction of information when the embedding dimension *m* increases by one [11, 21].

3 $$\begin{aligned} FuEn=\ln \phi ^m(r)-\ln \phi ^{m+1}(r) \end{aligned}$$

where,

4 $$\begin{aligned} \phi ^m(r)=\dfrac{1}{N-m}\sum _{i=1}^{N-m}\Bigg [\dfrac{1}{N-m-1}\sum _{p=1,p\ne q}^{N-m}D_{pq}^m\Bigg ]. \end{aligned}$$

The reduced entropy values are in accordance to the complexity loss theory of disease which attributes to reduced adaptive capabilities of individuals owing to the effect of the disease [22]. The parameters for the entropy calculation is generally selected as, $m=3$ and $r=0.2*std(y)$.

**Table 3:** Description and STAR classification of ataxic features

Test (device)	Feature	Description	STAR
FCT (Kinect©)	$AcAlt^{X,Y(L,R)}_{FCT}$	Acceleration alteration counts the number of times the acceleration is altered	Stability
	$ReTi^{X,Y(L,R)}_{FCT}$	Reaction time reflects the cross correlation of the two time sequences representing the marker and the target	Timing
	$KiDe^{(L,R)}_{FCT}$	Kinematic delay measures the ratio of the index of difficulty and the movement time	Timing
	$DTWEr^{X,Y(L,R)}_{FCT}$	Dynamic time warping based error measures the displacement between the performance marker and the target	Accuracy
FTT (IMU)	$CITI^{(L,R)}_{FTT}$	Coefficient of variation of inter-tap interval describes variability with respect to speed	Timing
	$FuEn^{AcX(L,R)}_{FTT}$	Fuzzy entropy describes the irregularity of the acceleration on X axis	Stability
	$FuEn^{AcZ(L,R)}_{FTT}$	Fuzzy entropy describes the irregularity of the acceleration on Z axis	Rhythmicity
	$FuEn^{GyX(L,R)}_{FTT}$	Fuzzy entropy describes the irregularity of the of gyroscopic measurement on X axis	Rhythmicity
FNT (IMU)	$RF^{AAcX(L,R)}_{FNT}$	Resonant frequency (RF) at the angular acceleration on X axis	Stability
	$RF^{AAcZ(L,R)}_{FNT}$	RF at the angular acceleration on Z axis	Stability
	$MR^{AAcX(L,R)}_{FNT}$	Magnitude at resonance (MR) at the angular acceleration on X axis	Stability
	$MR^{AAcZ(L,R)}_{FNT}$	MR at the angular acceleration on Z axis	Stability
	$RF^{AAcY(L,R)}_{FNT}$	RF at the angular acceleration on Y axis	Timing
	$MR^{AAcY(L,R)}_{FNT}$	MR at the angular acceleration on Y axis	Rhythmicity
DDKT (IMU)	$RF^{AngX(L,R)}_{DDK}$	RF of the angle on X axis	Stability
	$RF^{AngZ(L,R)}_{DDK}$	RF of the angle on Z axis	Stability
	$MR^{AngX(L,R)}_{DDK}$	MR at angle on X axis	Stability
	$MR^{AngZ(L,R)}_{DDK}$	MR at the angle on Z axis	Stability
	$RF^{AcX(L,R)}_{DDK}$	RF at the acceleration on X axis	Stability
	$RF^{AcZ(L,R)}_{DDK}$	RF at the acceleration on Z axis	Stability
	$MR^{AcX(L,R)}_{DDK}$	MR at the acceleration on X axis	Stability
	$MR^{AcZ(L,R)}_{DDK}$	MR at the acceleration on Z axis	Stability
	$RF^{AAcY(L,R)}_{DDK}$	RF at the angular accelerations on Y axis	Timing
	$MR^{AAcY(L,R)}_{DDK}$	MR at the angular accelerations on Y axis	Rhythmicity

#### Finger to nose test (FNT) and tests of dysdiadochokinesia (DDKT)

There are movement characteristics in FNT and DDKT that can be considered together in analysis. They are both repetitive movements that require a stable platform (shoulder in both cases). In both tests, ataxia is not only characterised by variability in rhythm but also by prolonged task duration resulting from displacement errors when moving. Such characteristics are amenable to investigation using frequency domain techniques. Measurements from the accelerometer and gyroscope were analysed in terms of the resonant frequency (RF) and its magnitude (MR) using Fast Fourier Transforms (FFT) with appropriate filtering parameters (6th order bandpass Butterworth filter with the cut-off frequency region of 2–5 Hz). In the FNT, the angular accelerations and linear accelerations can be effectively used to characterise ataxia [12]. The RF and MR of angular accelerations in the three axes were calculated as well as the linear acceleration in the $X$ axis. Only RF was applied to the linear acceleration in the $Y$ and $Z$ axes. In the case of the DDKT, RF and MR of angular acceleration and linear accelerations in all axes best distinguished between ataxics and controls [12].

### Statistical inferences

Statistically significant difference between ataxics and controls were identified using hypothesis tests. Normality in the variables was tested using the Shapiro–Wilk test. The t-Student’s test was applied for normally distributed variables. The Wilcoxon rank-sum test or Mann–Whitney U test was applied for variables that were not normally distributed. To test for validity, Spearman correlation was used to measure the relationship between objective measurements and clinical scales. The sample size used in this study was determined to detect the minimum effect size of 0.88 with 80% statistical power and significance level ($\alpha$) of 5%. Similarly, for testing correlation, the effect size (r) of 0.30 was used. The power analysis was performed using G*Power version 3.1.9.4 [23].

### Feature selection

The four tests of upper limb ataxia produced many features. As the feature space was unlikely to be uniformly populated, there was a risk of overfitting a learning model. To overcome this and improve the prediction power the number of features were reduced using feature selection (FS) techniques. The Feed Backward Feature Elimination (FBE) [24] was employed along with three other widely-used methods involving Random Forest [25], RELIEF [26] and LASSO [27]. The central idea of FBE is to find a subset of features that increases the model’s performance. In each iteration of the process, 90% of the data was randomly selected and a feature elimination decision was made using a threshold $\alpha$ (significance level) on the *p*-value of the feature (with the null hypothesis $H_0$ that the examining feature is independent of the predicted score given the set of currently selected features). Therefore, only features that significantly impacts the output (*p*-value $<\alpha$) are selected for the feature subset.

**Fig. 2:**
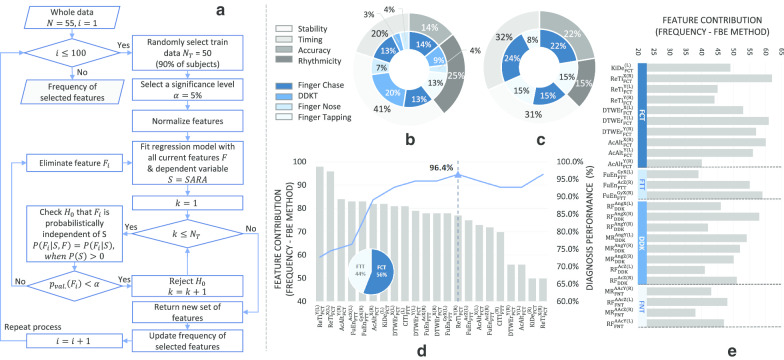
Feature selection and contribution.** a** FBE-based process of obtaining selection frequency of features.** b**,** c** STAR distribution of the selected features and test distribution in each partition:** b** All four tests and** c** FCT and FTT.** d** Feature contributions of FCT and FTT.** e** Feature contributions of the 4 tests (first 22 features)

In our experiment, we repeated the process 100 times to obtain the selection frequency of each feature in estimating its significance in the assessment/diagnosis problem. Details of the process were explained in the flowchart in Fig. 2a. Since the process is time consuming, computational performance was improved by employing the Parallel Computing Toolbox of MATLAB version 2019b to simultaneously execute the computations.

### Discrimination and severity analysis

Features with high selection frequency from the FBE were used to classify control and CA groups and predicting the severity of ataxia. Classification models for diagnosis included Linear Discrimination (LD) [28], Quadratic Discrimination Analysis (QDA) [29], Support Vector Machine (SVM) [30], K-Nearest Neighbour (KNN) [31]. Leave-One-Out (LOO) cross validation estimated the generalised performance of the diagnostic model as well as the severity predicting regression model [32]. The effectiveness of the model was evaluated through a number of statistical measurements including accuracy (ACC), F1-score, the stability of the model by the area under the Receiver Operating Characteristics curve (AUC), the sensitivity measure (or Recall), and Precision.

For regression analysis, we employed the Ridge regression method to correlate the proposed features with the SARA scores. This model avoids over-fitting when working with small data sets by forming a linear model to estimate the severity for the given input feature vector. In order to generate a general instrumented score, a severity scale that mirrored the SARA upper limb scores was developed. As there were not any ataxic subjects in the cohort who were rated with a SARA score of 4 (i.e all subjects ranged from 0 to 3), the instrumented severity scale was limited to 4 levels defined as follows:

- Level 0 : Normal, no dysmetria, tremor or irregularities.
- Level 1: Minimal dysmetria or low amplitude tremor or slight irregular motion.
- Level 2: Moderate, clear dysmetria, tremor or clearly irregular motion.
- Level 3: Severe, dysmetria in large range, high amplitude tremor or very irregular motion.

## Results

### Feature significance

**Table 4:** Mean, standard deviation, effect size measure and correlation coefficient values with SARA scores of the extracted features from CA subjects and controls

Test	Feature name	Unit	Subjects with CA $n = 41$	Controls $n = 14$	ES and *p*-value CA vs HC	CC and *p*-value SARA-Total	CC and *p*-value SARA-UL
FCT	$KiDe^{(L)}$	bits/s	2.683 ± 0.511	3.494 ± 0.431	1.646 (< 0.001*)	− 0.572 (0.003*)	− 0.663 (< 0.001*)
	$KiDe^{(R)}$	bits/s	2.517 ± 0.553	3.575 ± 0.352	2.070 (< 0.001*)	− 0.504 (0.010*)	− 0.380 (0.061)
	$ReTi^{X(L)}$	ms	1074 ± 292	761 ± 83	1.217 (< 0.001*)	0.610 (0.001*)	0.659 (< 0.001*)
	$ReTi^{X(R)}$	ms	1166 ± 332	740 ± 84	1.462 (< 0.001*)	0.358 (0.079)	0.223 (0.284)
	$ReTi^{Y(L)}$	ms	1076 ± 294	762 ± 85	1.211 (< 0.001*)	0.595 (0.002*)	0.641 (< 0.001*)
	$ReTi^{Y(R)}$	ms	1170 ± 334	743 ± 83	1.456 (< 0.001*)	0.360 (0.077)	0.228 (0.272)
	$DTWEr^{X(L)}$	px	1978 ± 648 (× 10)	1237 ± 182 (× 10)	1.299 (< 0.001*)	0.433 (0.031*)	0.394 (0.051)
	$DTWEr^{X(R)}$	px	2362 ± 853 (× 10)	1.201 ± 291 (× 10)	1.539 (< 0.001*)	0.305 (0.138)	0.086 (0.682)
	$DTWEr^{Y(L)}$	px	2277 ± 924 (× 10)	1.301 ± 253 (× 10)	1.202 (< 0.001*)	0.684 (<0.001*)	0.552 (0.004*)
	$DTWEr^{Y(R)}$	px	2705 ± 117 (× 10)	1.471 ± 250 (× 10)	1.209 (< 0.001*)	0.290 (0.159)	0.109 (0.604)
	$AcAlt^{X(L)}$	times	33.5 ± 12.5	22.1 ± 4.7	0.984 (< 0.001*)	0.522 (0.008*)	0.507 (0.010*)
	$AcAlt^{X(R)}$	times	35.7 ± 14.5	20.9 ± 2.9	1.163 (< 0.001*)	0.553 (0.004*)	0.271 (0.191)
	$AcAlt^{Y(L)}$	times	25.7 ± 11.7	16.1 ± 3.6	0.926 (< 0.001*)	0.275 (0.184)	0.317 (0.122)
	$AcAlt^{Y(R)}$	times	26.7 ± 12.1	14.1 ± 2.5	1.191 (< 0.001*)	0.202 (0.332)	0.105 (0.618)
FTT	$FuEn^{AcX(L)}$	nat	1.096 ± 0.321	1.422 ± 0.240	1.075 (< 0.001*)	0.345 (0.092)	0.117 (0.578)
	$FuEn^{AcX(R)}$	nat	1.147 ± 0.299	1.512 ± 0.348	1.175 (< 0.001*)	0.161 (0.442)	− 0.043 (0.837)
	$FuEn^{AcZ(R)}$	nat	0.914 + 0.257	1.088 + 0.344	0.621 (0.049*)	0.569 (0.003*)	0.397 (0.049*)
	$FuEn^{GyX(R)}$	nat	0.107 + 0.065	0.168 + 0.098	0.824 (0.019*)	0.138 (0.509)	− 0.068 (0.746)
DDKT	$RF^{AngY(R)}$	Hz	40.331 ± 15.166	23.212 ± 20.086	1.037 (0.002*)	− 0.017 (0.936)	− 0.033 (0.877)
	$MR^{AngX(R)}$	mV	10.945 + 10.046	6.675 + 7.174	0.453 (0.028*)	0.256 (0.216)	0.373 (0.066)
	$MR^{AngY(R)}$	mV	3.383 + 1.630	4.561 + 1.892	0.694 (0.029*)	− 0.328 (0.110)	− 0.292 (0.157)
	$RF^{AcZ(L)}$	Hz	1.900 + 1.245	2.187 + 1.104	0.237 (0.049*)	− 0.322 (0.116)	− 0.220 (0.291)
	$MR^{AcX(L)}$	mV	2.026 + 1.395	2.675 + 1.774	0.433 (0.033*)	− 0.133 (0.527)	− 0.068 (0.748)
	$MR^{AcX(R)}$	mV	1.965 ± 1.225	3.492 ± 2.023	1.045 (0.002*)	− 0.031 (0.885)	0.104 (0.621)
	$MR^{AcZ(L)}$	mV	1.413 ± 0.441	1.892 ± 0.375	1.126 (< 0.001*)	− 0.481 (0.015*)	− 0.354 (0.083)
	$MR^{AcZ(R)}$	mV	1.550 ± 0.551	1.964 ± 0.399	0.800 (0.002*)	− 0.345 (0.093)	− 0.148 (0.481)
FNT	$RF^{AAcX(R)}$	Hz	3.289 ± 2.353	5.721 ± 3.417	0.916 (0.004*)	0.097 (0.644)	0.036 (0.866)
	$MR^{AAcX(L)}$	mV	5.151 ± 3.174	8.022 ± 3.018	0.915 (0.003*)	− 0.295 (0.152)	− 0.283 (0.170)
	$MR^{AAcY(L)}$	mV	7.879 ± 4.558	15.824 ± 5.116	1.690 (< 0.001*)	− 0.429 (0.032*)	− 0.483 (0.015*)
	$MR^{AAcY(R)}$	mV	8.809 + 5.296	12.805 + 5.122	0.761 (0.014*)	− 0.369 (0.069)	− 0.344 (0.092)
	$MR^{AAcZ(L)}$	mV	3.729 + 2.417	5.646 + 3.215	0.727 (0.028*)	− 0.359 (0.078)	− 0.467 (0.022*)

Data are shown in mean ± standard deviation

*CA* subjects with cerebellar ataxia, *HC* controls, *ES* effect size, *CC* correlation coefficient (Spearman)

*p-value < 0.05

Table 4 shows the 31 (out of 62) objective features generated during the performance of FCT, FTT, FNT and DDKT that reached statistical significance ($p < 0.05$). These features represent movement characteristics of ataxic subjects that differ significantly from controls. Movements performed by subjects with CA were significantly slower (e.g. *KiDe*, $p < 0.001$) with longer reaction times (e.g. *ReTi*, $p < 0.001$) suggesting that a longer time is required to recognise the new target and react. The movements of controls were relatively more complex movements (entropy measures, $p < 0.05$) than movements of ataxic subjects and had less functional variability (e.g. *DTWEr*, $p < 0.001$). There were measures in the non-primary axis whose values in controls differed significantly from ataxic subjects. These difference most likely arose from instability in proximal or stabilising joints of individuals with CA. The clinical validity of these measures was assessed by correlating with the SARA scores (SARA-Total and SARA-UL: see last two columns of Table 4). Some FCT movement characteristics were moderately ($p < 0.01$) to significantly ($p < 0.001$) correlated with the SARA ratings. Entropy features of FTT correlated moderately with SARA, while DDKT and FNT features correlated weakly with SARA ($p < 0.05$).

### Selection frequency of features

Figure 2e shows the selection frequency following 100 iterations of the FBE process applied to 22 features with the highest contribution (first 22 features) in the combined test model. The frequency implies the contribution of each feature in estimating severity of ataxia. Higher selection frequency, implies a greater possibility of the feature’s selection in the final subset. Of note, FCT provided more important features (including *ReTi*, the feature with the highest selection frequency) than other tests and all FCT movement features appear in the chart. As discussed later this reflects the importance of FCT’s contribution to the objective ataxia score. In comparison, FNT contributed the least to the selected feature subset. Despite fewer features (3), FTT features were selected with higher frequency than DDKT or FNT related features.

**Fig. 3:**
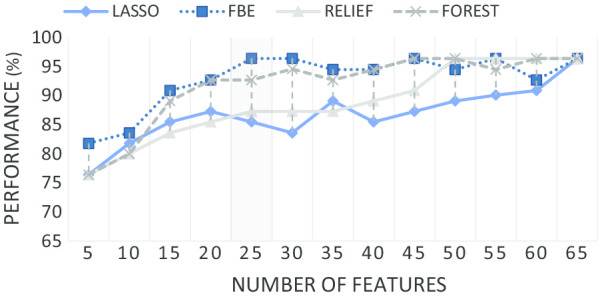
Classification performance of the 4 feature selection methods

Figure 3 plots the classification performance (Y axis) of the four feature selection methods against the number of selected features (X axis). Here, the aim was to find the smallest subset of features that produced a high performance (accuracy) in diagnosing CA. FBE outperformed LASSO, RELIEF and Random Forest, providing a 96.4% accuracy with the first 22 of the 64 (top 34%) features with the highest selection frequency. The accuracy was low (ACC 83%) with the first 5 features. As the number of features increased, the performance of FBE fluctuated around 95.4% (std. ± 1.4%) with a similar performance to other methods. List of selected features are shown in the bar chart of Fig. 2e.

**Table 5:** Experimental results of different combination of feature selection and binary classification methods

Classifier	FS	Recall	Precision	MCC	ACC	AUC
QDA	*FBE*	*0.98*	*0.98*	*0.90*	*96.4*	*0.97*
	*RF*	*0.93*	*0.98*	* 0.81*	*92.7*	*0.96*
	RELIEF	0.93	0.90	0.66	87.3	0.88
	LASSO	0.85	0.90	0.54	81.8	0.87
LD	FBE	0.90	0.93	0.67	87.3	0.85
	RF	0.85	0.90	0.54	81.8	0.80
	RELIEF	0.88	0.78	0.19	72.7	0.65
	LASSO	0.83	0.79	0.20	70.9	0.82
SVM	FBE	0.83	0.92	0.57	81.8	0.85
	RF	0.88	0.92	0.64	85.5	0.89
	RELIEF	0.90	0.97	0.78	90.9	0.95
	LASSO	0.90	0.93	0.67	87.3	0.90
KNN	*FBE*	*0.95*	*0.98*	*0.86*	*94.5*	*0.97*
	RF	0.90	0.93	0.67	87.3	0.90
	RELIEF	0.88	0.88	0.52	81.8	0.93
	*LASSO*	*0.95*	*0.95*	*0.81*	*92.7*	*0.94*

**Table 6:** Performance of classification models to distinguish CA subjects from controls from features of individual test and of combined tests

Test	Recall	Precision	MCC	ACC	AUC
FCT	0.95	0.95	0.81	92.7	0.98
FNT	0.83	0.92	0.57	81.8	0.80
FTT	0.88	0.92	0.64	85.5	0.82
DDKT	0.90	0.92	0.78	90.9	0.96
All tests	0.98	0.98	0.90	96.4	0.97

### “Diagnosis” of ataxia and SARA based Severity Estimation

**Fig. 4:**
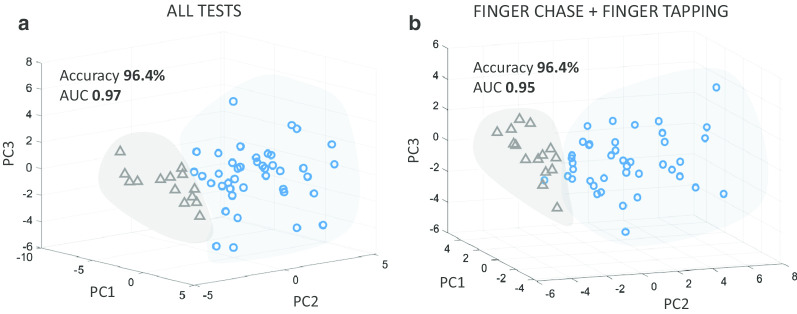
Group classification in PCA.** a** All features.** b** FCT and FTT features

The accuracy of the system in making a binary diagnostic classification (into ataxics and controls) can be considered with Precision and Recall values. Precision is measured by expressing the number of correctly identified ataxic subjects as a fraction of the number of identified ataxic subjects. Recall expresses the number of correctly identified ataxic subjects as fraction of the total number of actual ataxic subjects. Therefore, the closer a mode’s Precision and Recall are to 1, the more effective the model is in sorting (“diagnosing”) ataxic subjects from controls. The effect of the higher number of ataxic subjects on the model’s accuracy was assessed using Matthew’s Correlation Coefficient (MCC). The MCC ranges from − 1 to 1, where 1 depicts a perfect prediction. The diagnostic performance of four learning models (QDA, LD, SVM, KNN) and four feature selection methods (FBE, LASSO, RELIEF, RF) were compared (Table 5). The QDA + FBE pair outperformed the others in diagnostic performance with a greater accuracy (ACC 96.4%, Recall 0.98, Precision 0.98) and reliability (AUC 0.97 and MCC 0.90). This classification can be visualised by plotting the first three principle components of a Principal Component Analysis (PCA) (Fig. 4a). In summary, ataxic subjects can be identified (diagnosed) from controls with a high degree of accuracy [ACC > 92%, Precision and Recall > 0.9, MCC > 0.7 (Table 5)] using several models (QDA + FBE, QDA + RF, KNN + LASSO, KNN + FBE) generated from extracted features.

QDA provided a flexible decision boundary for assessing the influence of each clinical test on the capacity to accurately separate ataxic subjects from controls. Table 6 shows that FCT performed best (ACC 92.7%, Recall 0.95, Precision 0.95), despite fewer rhythmicity features in the selection (Table 4). Notwithstanding FCT’s performance, combining all tests provided a model with greater accuracy in “diagnosing” ataxic subjects (ACC 96.4% compared to ACC 92.7%, Table 6) and less affected by the imbalance between ataxic and control subjects (MCC 0.90 compared to MCC 0.81, Table 6). This superior performance of the combined tests implies that a contribution from all domains is required for best “diagnostic” performance: rhythmicity features missing from FCT were provided by other tests. However, domains can be provided by more than one test; for instance, rhythmicity is contributed by FTT, FNT and DDKT and stability is provided by all 4 tests. This raises the question whether all tests are required to accurately assess the severity of ataxia. Thus, we mapped features and tests to the STAR dimensions and then different combinations of tests are investigated in the last subsection.

**Fig. 5:**
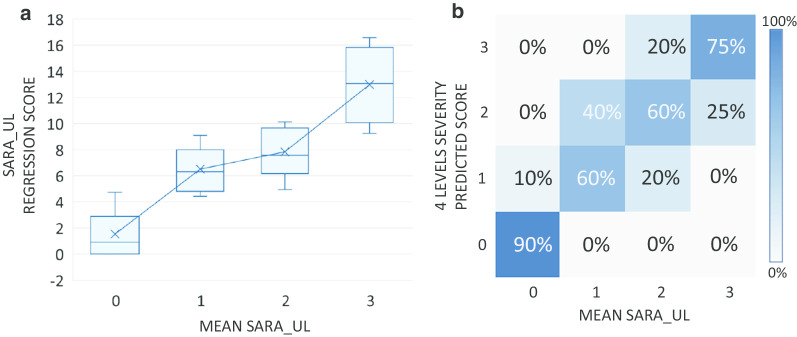
Severity estimation.** a** Distribution between regression scores and mean upper-limb SARA scores.** b** Severity agreement between the 4-level predicted scores and the mean upper-limb SARA scores

As previously shown, the values predicted by the model were highly correlated with the mean SARA score. The predicted scores, *ps* (all tests, Table 9), of the subjects were plotted against their corresponding mean SARA scores (the rounded average of the scores of the upper limb tests: mean $SARA\_UL$) in Fig. 5a). The boxplots represent the distribution of the *ps* in each of the four severity levels (0–3—see "Methods"). For comparison, the predicted scores were classified into the 4-level scale as follows: $ps<4$ belongs to the normal group (level 0), $4\le ps<7$ belong to the mild group (level 1), $7\le ps < 10$ belongs to the moderate group (level 2) and $ps\ge 10$ belongs to the severe group (level 3). The agreement matrix in Fig. 5b outlines the mapping of the predicted scores into each clinical severity levels. In particular, subjects scored ‘0’ by SARA can be predicted with a high degree of accuracy (90%) from the underlying system. No subject scored ‘0’ or ‘1’ by SARA was classified to moderate (level 2) nor to severe group (level 3) by the model and no subject scored ‘1’/‘2’/‘3’ by SARA was classified as normal (level 0) by the model.

**Table 7:** Common selected feature in each test from the 4 FS methods

	FCT	FTT	FNT	DDKT
S	n/a	n/a	$MR^{AAcX}_{FNT}$	$RF^{AngX}_{DDK}$
			$RF^{AAcY}_{FNT}$	$MR^{AngX}_{DDK}$
			$RF^{AAcZ}_{FNT}$	$MR^{AngZ}_{DDK}$
T	$KiDe^{X,Y}_{FCT}$	$CITI_{FTT}$	$RF^{AAcY}_{FNT}$	n/a
	$$ReTi^{X,Y}_{FCT}$$			
A	n/a	n/a	n/a	n/a
R	n/a	$FuEn^{GyX}_{FTT}$	n/a	n/a

*n/a* not available

**Table 8:** Statistical measurement of regression analysis of features from each dimension in STAR with SARA scores

	Statistical measure	SARA-total	SARA-UL (sum)	SARA-UL (mean)
S	Agreement (%)	–	–	57%
	R-squared	0.52	0.61	0.62
	Corr. coef.	0.6	0.69	0.69
T	Agreement (%)	–	–	75%
	R-squared	0.64	0.82	0.84
	Corr. coef.	0.77	0.87	0.85
A	Agreement (%)	–	–	56%
	R-squared	0.48	0.54	0.61
	Corr. coef.	0.6	0.55	0.52
R	Agreement (%)	–	–	40%
	R-squared	0.33	0.43	0.39
	Corr. coef.	0.35	0.47	0.38

### Disability association to The STAR dimensions

The extracted features were assigned to one of the proposed Holmesian dimensions (STAR) of ataxia. Details of this clustering are presented in Table 3. Selected features in each dimension of the STAR together with their contribution to the feature selection process can also be related to the presence and severity of ataxia. Using this approach, it is possible to attribute the contribution of each STAR dimension to the overall diagnosis of ataxia (Fig. 2a), with the stability features contributing most (41% compared to 25% from Rhythmicity, 20% Timing and 14% Accuracy). Most of the stability features were derived from the DDKT and FNT.

Considering dimensional aspects of the extracted features, Table 8 records the correlation between the STAR features and the three SARA scores, i.e. the SARA-Total and the SARA-UL (in terms of sum and mean). Features corresponding to timing provided the highest correlation with the SARA scores (0.77, 0.87 and 0.85) whereas features corresponding to rhythmicity had the lowest correlations (0.35, 0.47 and 0.38).

### Combination of tests

In order to determine whether SARA scores can be predicted with fewer clinical tests, the performance of different test combinations was investigated. As discussed in the STAR analysis, only the FCT provided features that corresponded to the accuracy dimension. Assuming that all STAR dimensions of ataxia will be required for the best prediction of SARA, the presence of FCT features will be essential. The test groupings are considered as follows:

- Group 1 (G1): FCT and FTT
- Group 2 (G2): FCT and FNT
- Group 3 (G3): FCT and DDKT
- Group 4 (G4): FCT and FNT and DDKT
- Group 5 (G5): FCT and FTT and DDKT
- Group 6 (G6): FCT and FTT and FNT

**Table 9:** Statistical measurements of binary classification and SARA scores correlation from different combination of tests

Group	ACC (%)	AUC	Recall	Precision	F1-score	CC
G1	*96.4*	*0.97*	*0.95*	*1.00*	*0.97*	0.80*
G2	85.5	0.95	0.95	0.89	0.92	0.40
G3	76.4	0.70	0.98	0.75	0.85	0.36
G4	87.3	0.89	0.95	0.82	0.88	0.48
G5	92.0	0.90	0.98	0.93	0.95	0.53
G6	92.7	0.92	0.95	0.95	0.95	0.60*
All tests	96.4	0.96	0.98	0.98	0.98	0.68*

*CC* Correlation coefficient (Spearman)

*p-value < 0.05

The highest accuracy in sorting ataxics from control subjects was provided by Group 1 (Table 9). This combination also performed best in terms of AUC, sensitivity, and precision. Clear separation between ataxic and control subjects is evident in Fig. 4b. Additionally, the classification achieved by Group 1 was similar to that achieved by the combination of all tests, but with a greater effect size of the correlation. The contribution of each STAR Domain to Group 1 is 32% timing, 31% stability, 22% accuracy and 15% rhythmicity (Fig. 2c). Sensitivity and accuracy are important for diagnostic accuracy and F1-score, which is the harmonic mean of the sensitivity and precision [33]. The F1-score of the combined tests (0.98) was marginally better than Group 1’s F1-score (0.97). However, the correlation between the scores predicted by the regression model and the SARA-Total (Table 9), was higher for Group 1 (0.8) than the combined tests (0.68), coefficient of 0.8 compared to 0.68 when using all tests). Therefore, in the instrumented system, the FCT and FTT combination provided the best agreement with the clinical assessment of ataxia in the upper limb.

## Discussion

Previous studies have shown that each individual bedside test can be emulated using features derived from sensors worn while the bedside tests of CA were being performed [3, 11, 12]. However, each test emphasises different STAR domains and thus begs the questions of which are the most useful in identifying ataxia and how much redundancy is there in these tests. This was achieved in this study by obtaining instrumented data while four bedside tests (FCT, FTT, FNT and DDKT) were performed and features from these data were used to model the SARA-Total and the SARA-UL scores. Approximately half of the features were significantly correlated with the two SARA scores with the highest correlation of individual features being 0.68 with the SARA-Total and 0.66 with the SARA-UL (Table 4). The feature set was further refined a smaller subset of 22 features that maintained a high performance (accuracy) in sorting ataxics from controls (Fig. 3). Using several different learning models it was possible to identify (diagnose) ataxics accurately using these 22 extracted features (Table 5).

Not all bedside tests contributed equally to the performance of these models. FCT contributed the most features as well as the most frequently selected features (Fig. 2e). FCT combined with FTT provided enough features to performs as well as the combined feature set (Fig. 4). One conclusion is that FCT was necessary because it was the only test that included the accuracy domain from STAR (Table 3). This may be in part self-fulfilling and reflect aspects of the STAR criteria but future studies exploring different definitions of accuracy or other tests which measure accuracy could address this issue. Even though accuracy was only present in FCT, features related to the accuracy dimension were not selected in the list of common features (Table 7). One possible explanation is that accuracy is highly correlated with timing features which may in turn have contributed to the exclusion of this dimension in LASSO. It is also noteworthy that kinematic delay in FCT contributed the most to the performance of the two models and the number of timing features significantly increased and were the highest proportion of features when FCT and FTT were combined (32% in Fig. 2c compared to 20% in Fig. 2b). Further, the predicted values from the regression model that used timing features demonstrated the highest correlation with the SARA scores (Table 8) indicating the important role of timing in the clinical assessment of ataxia. Consistently selected features were obtained by extracting common features from the four FS methods. It also should be noted that features belonging to the timing domain were consistently selected from 3 out of 4 tests. They were also significant features in the combined model of G1 (Fig. 2d) and all the tests model (Fig. 2e).

Another conclusion from this study is that there is redundancy in the bedside tests and not all are required to identify the presence and severity of ataxia. Multiple tests generated a plethora of features, each representing aspects of ataxic movements but also likely containing redundancy. The performance analysis of subsets of the tests uncovered the optimal combination of information that essentially led towards the reduction of tests. Different groupings result in feature combinations that can improve or decrease the performance of learning models (Table 9) and decreasing the number of features without affecting the performance of the learning model infers that redundant information has been removed. Combination of FCT and FTT alone did not degrade diagnostic performance (Fig. 4) and slightly improved correlation in severity estimation in comparison to the performance of all tests combined (Table 9). On the other hand, the FCT and DDKT combination was the lowest accuracy in identifying ataxia.

While the SARA prescribes that the examiner should evaluate the (a) accuracy in reaching target in the FCT; (b) the speed or time required to perform the DDKT; (c) the amplitude of the kinetic tremor in the FNT, clinical assessment is blind in what features are found to best correlate with SARA scores. It is thus of interest that not only features that clinicians are explicitly directed to assess (e.g. accuracy in the FCT) were captured but there were also added features, e.g. initiation delay. As the instrumented test depends on these features to accurately model the SARA, this extra information is presumably identified and accounted for (possibly subconsciously) by an experienced clinician even if it is not part of their explicit evaluation. Despite stability, timing, accuracy and rhythmicity being dependent on each other as discussed in [34], in our study, we referred to the SARA to assess a range of different impairments which are related to each STAR dimensions. Further research is required to assess the interdependency of each of the STAR dimensions.

Features could be sorted into the four ataxia dimensions (STAR). This was most straightforward in the case of FCT, whose features could be readily placed into a STAR domain according to its physical meaning. In the case of features from FTT, FNT and DDKT, their attribution to a specific STAR domain was according to whether the feature was more related to the primary or the secondary axis of movement. The former corresponds to movements along the direction of the axis most related to accomplishing the task objectives, e.g. the upward/downward movement in tapping or the rotation of the forearm in DDKT (Fig. 1). Secondary-axis movements mostly occur because of instability of the execution platform, i.e. the proximal joints (shoulder or elbow) which must be stable for accuracy of the moving distal hand or wrist. Therefore, significant differences in secondary axes motion in ataxic and control subjects were attributed to instability in this platform. Due to the factor of repetition, the primary movement is required to adhere to a self-defined rhythm [6]. Measures pertaining to this axis can be used to infer the deficits in rhythmicity or timing. In the frequency analysis, timing aspects or “how quick is the movement performed” were described by the RF, whereas MR indicated the intensity of the rhythmic movement [12] which was considered as a measure of rhythmicity.

Learning models will always be improved with more subjects. Nevertheless, a cohort of people with CA of this size is relatively large in comparison to earlier studies of ataxia [13, 15, 35]. Furthermore, power analysis and rigorous cross validation process validated the reliability and statistical significance necessary for assertions of clinical validity. There is an assumption that “all cerebellar ataxia is the same” and it is possible, indeed likely, that the presence of somatosensory impairment, vestibular involvement or other central nervous system (CNS) lesions may affect objective assessment of ataxia. One of the motivations for producing more precise means of assessing ataxia is to establish whether the factors that might differentiate ataxia associated with other neural lesions might differ from “pure” cerebellar ataxia. This would be a subject of future studies. In a similar vein more severe ataxia reflected by SARA scores $>3$ would be important in future studies. Another potential direction of research would explore the combination of FCT and FTT as a mechanism of capturing the progression of disease in a longitudinal study. With the rapid advancement in pervasive Internet-of-Things technologies, capturing the severity of CA subjects more regularly in their natural environment (non-clinical setting) and monitor the progress remotely will inevitably enable more personalized health care with effective rehabilitation programs.

## Conclusion

The instrumented assessment scheme proposed was based on the four widely-used motor tests of upper limb functionality. The system described here was able to support clinical decision making with a fewer number of features selected from the conventional execution of these tests. The features were grouped and evaluated through the proposed definition of the ataxic manifestations (STAR) in a quantitative form which provided plausible interpretation of ataxia. In the scope of upper limb assessments, the characteristics belonging to timing resulted in the highest association with the SARA total score. A 4-level discrete form of severity rating scale was introduced to be in line with the conventional scale, the SARA. This further confirmed the agreement with the current practice of clinical assessments and provided a severity estimation within acceptable levels of deviations. The other important finding of this study is that the FCT and FTT were identified as the most suitable combined assessments that presented highly accurate CA diagnosis and severity estimation, among other combinations of tests. The reduction of tests would potentially lead to a more cost-effective assessment strategies to be performed in clinical practices where resources such as clinician time and the number of patient visits are often limited.

## Data Availability

The data that support the findings of this study are available from the corresponding author upon reasonable request.

## Abbreviations

CA: Cerebellar ataxia
FCT: Finger chase test
FTT: Finger tapping test
FNT: Finger to nose test
DDKT: Tests of dysdiadochokinesia
ISULA: Instrumented System for the objective assessment of Upper Limb Ataxia
SARA: Scale for the Assessment and Rating of Ataxia
SARA-UL: Total score of upper limb tests in SARA
SARA-Total: Total scores of all tests in SARA
STAR: Manifestations of ataxia (stability, timing, accuracy, rhythmicity)
FBE: Feed backward feature elimination
LASSO: Least absolute shrinkage and selection operator
QDA: Quadratic discrimination analysis
RR: Ridge regression
SVM: Support Vector Machine
KNN: K-Nearest Neighbour
LD: Linear Discrimination
LOO: Leave-One-Out cross validation
IMU: Inertial measurement unit
FFT: Fast Fourier Transform
ACC: Accuracy
AUC: Area under the Receiver Operating Characteristics curve
MCC: Matthews correlation coefficient
ES: Effect size
CC: Spearman correlation coefficient
CNS: Central nervous system
